# On the Diagnostic and Prognostic Value of Tendon Reflexes

**Published:** 1892-09

**Authors:** John Furguson

**Affiliations:** M. A., M. D. (Tor.), L. R. C. P. (Edin.)


### ON THE DIAGNOSTIC AND PROGNOSTIC VALUE OF TENDON REFLEXES.

BY JOHN FURGUSON, M. A., M. D. (TOR.), L. R. C. P. (EDIN.).

The physiology of the tendon reflexes is a matter of much dispute. Some maintain that they are of a reflex character and of spinal origin, while on the other hand, equally competent observers consider that they are due to a mechanical excitation of the muscle, such as a blow upon its tendon.

But there is not such a great difference between these views, when both agree that the state of muscular irritability which causes the muscle to respond to excitation, is due to a nerve-stimulus supplied to it from the spinal cord. The writer believes that the tone of the muscle is due to its normal and healthy connection with the spinal centre; and this latter receives its stimulus from the cerebellum, the cerebellar influx, and is held in check by the cerebrum, the cerebral inhibition. Derangement of this mechanism would result in either a deficiency, an exaggeration, or an abolition of the reflexes.

When the control of the cerebrum is removed by the occurrence of a hemorrhage, the growth of a tumor, the existence of a degeneration, or an epileptic paroxysm, the muscular irritability increases, and the tendon reflexes are exaggerated. In cases of cerebral hemorrhage, the tendon reflexes may be enfeebled or abolished, provided the apoplexy was of sudden origin. No special prognostic value can be attached to the absence of the deep reflexes in such cases, as the real causes of the abolition of the tendon reflexes are the position and the suddenness of the hemorrhage rather than its extensive character. In the great majority of cases of cerebral hemorrhage, the reflexes soon return. If there is any pressure on the cerebellum, however, the reflexes will not return as long as this pressure lasts. In cases of cerebellar tumor, the knee-jerk is usually wanting, and cerebellar titubation may be present.

In cases of injury to the spinal cord where the reflexes are absent after a lapse of several weeks, the prognosis is very grave, as the indications are that the cord lesion is a very serious one. In all diseases or injuries of the motor areas of the brain, causing a loss of control over the spinal cord; in all injuries and diseases of the motor paths, capsular, crustal, or pyramidal, by which the influence of the cerebral cortex is cut off from the spinal centres, there is developed a condition of extreme excitability of the reflexes. Such conditions as spastic paralysis following some lesion in the motor tracts, primary spastic paraplegia, cerebral spastic paralysis of children, the ataxic paraplegia of Gowers, and multilocular sclerosis, are all conditions in which we meet with an exaggerated condition of tendon reflexes. The sooner the knee-jerk becomes increased in these cases, and the greater the degree of this increase, the worse must the prognosis be. After a careful study of a number of cases of general paresis, the writer concludes:

1. When the knee-jerk is little affected, the disease in the cerebrum is not very extensive; and the more rapidly the disease increases, the more rapid will be the increase in the knee-jerk.

2. When the knee-jerk is reduced or lost, there is coincident disease in the spinal cord, either of the postero-external columns, or of the anterior cornua.

3. If the disease be in the posterior columns, there will be symptoms pointing to this, as found in tabes dorsalis.

4. When the cord disease is in the anterior cornua, along with the loss of the knee-jerk, there will be a flaccidity, atrophy, and degeneration of the muscles.

5. The loss of the knee-jerk, due to either of these morbid changes, would call for a very unfavorable prognosis, and would preclude much hope of even a temporary arrest of the disease.—*Medical Record*, New York.

